# Foreign Body Ingestion Experience in a Tertiary Hospital

**DOI:** 10.7759/cureus.86131

**Published:** 2025-06-16

**Authors:** Renad Aljohani, Muhannad N Almajed, Musa Khormi, Ahmed Alawfi, Wadha M Aldosary, Husam S Islam, Mohammed Hamaid, Dalia Albagli

**Affiliations:** 1 Pediatrics, Dr. Soliman Fakeeh Hospital, Jeddah, SAU; 2 Pediatric Gastroenterology, King Saud Medical City, Riyadh, SAU; 3 Pediatrics, King Saud Medical City, Riyadh, SAU

**Keywords:** child safety, endoscopic removal, foreign body ingestion, parental supervision, pediatric emergency

## Abstract

Background

Foreign body ingestion is a common pediatric emergency that can lead to significant morbidity. Understanding the patterns, management approaches, and outcomes of these cases is crucial for optimal patient care. This study aims to analyze the prevalence, management strategies, outcomes, and associated factors of foreign body ingestion cases in children presenting to the King Saud Medical City over a five-year period.

Methods

This analytical retrospective chart review study examined medical records of 203 pediatric patients (aged 0-18 years) who presented with foreign body ingestion between January 2018 and December 2023. Data were analyzed using descriptive statistics and chi-square tests.

Results

The majority of patients were aged 3-5 years (44.3%) and male (59.1%). Coins were the most commonly ingested objects (50.2%), followed by batteries (18.2%). Most foreign bodies were located in the esophagus (61.1%). Endoscopic removal was the primary intervention (70.9%), with a successful removal rate of 81.8%. Playing with small objects (84.7%) and a lack of supervision (75.4%) were the most common risk factors. Complete resolution was achieved in 95% of cases, with 70.4% requiring medical intervention.

Conclusions

Foreign body ingestion in children shows a favorable prognosis when managed appropriately. Early detection and intervention, primarily through endoscopic removal, lead to successful outcomes. Prevention strategies should focus on parental supervision and limiting access to small objects, particularly in children aged 3-5 years.

## Introduction

Ingestion of foreign bodies is quite common in children and can lead to multiple complications, such as bowel obstruction and perforation. In the United States alone, there have been over 100,000 reported cases [1]. Children under four years of age account for up to 75% of these cases [2]. A recent study conducted in Al Ahsa Eastern Province, Saudi Arabia, examined 91 pediatric patients with foreign body ingestion and found that 52.7% were less than three years old, while only 15.4% were six years or older [3]. Interestingly, the study also revealed that a higher percentage of male children (62.2%) had ingested foreign bodies compared to females (37.8%) [3].

Children most commonly ingest small batteries, fish bones, magnets, pins, and coins [4,5]. A study of 585 Omani children found that coins were the most frequently ingested item (41.3%), followed by disc batteries (12.2%) [6]. In approximately 80%-90% of ingestion cases, these small foreign bodies pass through the feces without any complications [7]. However, up to 10% of foreign bodies may remain in the gastrointestinal (GI) tract and require endoscopy-based removal. In 1% of cases, surgical intervention may be necessary to remove these foreign bodies [5,8].

Generally, when pediatric patients ingest foreign bodies, the prognosis is favorable and does not require medical intervention. However, serious complications and even death can occur when certain objects, such as small batteries or magnets, are ingested [5,9]. Children often present with various symptoms, including vomiting, dysphagia, a sensation in the esophagus, and odynophagia, after ingesting foreign bodies. The symptoms can also vary depending on the position of the foreign body. If a foreign body becomes lodged in the esophagus, it can cause cough, difficulty breathing, dysphagia, and sialorrhea.

In contrast, foreign bodies in the stomach are usually asymptomatic, although large objects can cause food refusal and vomiting. However, foreign bodies in the intestine can lead to complications such as ileocecal valve retention, perforation, and peritonitis [2,10]. A study conducted on Turkish children under the age of five found that the primary complaints were difficulty in swallowing (37%), cough (12%), vomiting (5%), stomach ache, and vomiting blood (2%) [11].

Various techniques can be used to investigate foreign bodies inside the body. X-ray is the preferred technique, as it can accurately determine the size, number, and location of the foreign bodies. In cases where X-rays are not helpful due to radiopaque objects, other imaging techniques, such as ultrasonography or magnetic resonance imaging (MRI), can be used to locate the position of the foreign bodies [10,12]. A recent study on Saudi children revealed that out of 117 patients, 92.9% only underwent radiographs to detect foreign bodies. Contrast swallow technique was used in 3.5% of the patients, and computed tomography (CT) was performed in 0.9% of the cases [13].

Initial assessment and clinical diagnosis are based on physical examination, symptoms, medical history, and the timing of presentation [4,5]. Endoscopy is the preferred procedure for removing foreign bodies, as it is minimally invasive. Endoscopic removal or spontaneous passage of the foreign bodies is the most suitable option. However, a study conducted in Saudi Arabia found that all three children who ingested magnets required surgical removal, with two undergoing open laparotomy to remove the magnet pieces from their GI tracts [14]. Recent advancements in upper GI tract endoscopy have made foreign body removal easier and safer for children. However, certain criteria should be considered when performing endoscopy in children, such as the time interval since ingestion, time of last meal, age, body weight, clinical symptoms, and the location of the foreign bodies in the GI tract [15].

## Materials and methods

Study design and setting

The study used an analytical retrospective chart review design to analyze the patterns, management, and outcomes of foreign body ingestion cases in pediatric patients at King Saud Medical City. This design allowed us to examine medical records comprehensively and identify trends in patient presentation, treatment approaches, and clinical outcomes.

The study was conducted at King Saud Medical City in Riyadh, Saudi Arabia, a tertiary care hospital that serves as a major referral center in the region, offering specialized care in various medical fields, including pediatrics. Data collection involved a systematic review of medical records, focusing on cases of foreign body ingestion in children. Patient selection was based on specific inclusion criteria to ensure comprehensive representation of foreign body ingestion cases across different age groups and presentation types.

Study population

The study employed a non-probability convenience sampling technique to include pediatric patients who presented to the emergency department of King Saud Medical City with foreign body ingestion between January 2018 and December 2023. We included patients aged 0 to 18 years and excluded those with incomplete medical records and cases where the final diagnosis was different from foreign body ingestion. This sampling approach allowed us to efficiently collect data from all available cases that met our inclusion criteria.

Data collection

Using a comprehensive and methodically designed data collection instrument that underwent face validation by two experts in pediatric emergency medicine, we systematically extracted detailed information from patient medical records. The collected data encompassed multiple domains, including comprehensive participant demographics, detailed medical histories, specific diagnostic techniques employed, step-by-step management approaches implemented, clinical outcomes observed throughout the treatment process, and various potential risk factors that may have contributed to the foreign body ingestion incidents.

Statistical analysis

Data were analyzed using Statistical Package for Social Sciences (SPSS) version 29 (IBM Corp., Armonk, NY). Descriptive statistics were calculated and presented as frequencies and percentages for categorical variables to provide a clear overview of the distribution patterns. To investigate potential relationships within the dataset, statistical associations between categorical variables were examined using chi-square tests, with Fisher's exact tests being employed when cell counts were insufficient for chi-square analysis. The threshold for statistical significance in all analyses was established at p<0.05.

Ethical considerations

The study protocol received approval from the Institutional Review Board at King Saud Medical City (IRB number: H1RI-26-Sep23-01). All data collected from patient medical records were handled with strict confidentiality, anonymized during analysis, and used solely for research purposes. Patient privacy was maintained throughout the study in accordance with institutional guidelines and ethical standards for retrospective research.

## Results

Our study included 203 pediatric patients who presented with foreign body ingestion at King Saud Medical City. According to Table 1, the demographic analysis revealed that the majority of patients (90, 44.3%) were between 3 and 5 years old, followed by children ≤2 years (65, 32.0%) and children >5 years (48, 23.6%). Male children represented a higher proportion of cases (120, 59.1%) than females (83, 40.9%). The vast majority of patients were accompanied by their parents (200, 98.5%), with very few cases involving grandparents (1, 0.5%) or nurses (2, 1.0%).

**Table 1:** Demographic characteristics N=203

Variable		N	%
Age (year)	≤2	65	32.0
	3-5	90	44.3
	>5	48	23.6
Gender of the child	Male	120	59.1
	Female	83	40.9
Guardian relationship to the patient	Parent	200	98.5
	Grandparent	1	0.5
	Nurse	2	1.0

As shown in Table 2, 110 cases (54.2%) were reported as emergencies, which required immediate care. Coins were the most commonly ingested foreign body (102, 50.2%), followed by batteries (37, 18.2%) and food bolus (15, 7.4%). The most frequent symptoms reported were vomiting (99, 48.8%), cough (40, 19.7%), and dysphagia (24, 11.8%), while 64 patients (31.5%) were asymptomatic. The foreign bodies were most commonly located in the esophagus (124, 61.1%), followed by the stomach (36, 17.7%) and the intestine (22, 10.8%).

**Table 2:** Medical history N=203. Examples of other types of ingestion: Pieces of paper, hair clips, and rubber bands.

Variable		N	%
Foreign body ingestion incidents reported as emergency case		110	54.2
Type of ingestion	Button batteries	37	18.2
	Coins	102	50.2
	Food bolus	15	7.4
	Magnet	10	4.9
	Screws	8	3.9
	Pins	4	2.0
	Toys or parts of a toy	3	1.5
	Other	24	11.8
Symptoms	Vomiting	99	48.8
	Dysphagia	24	11.8
	Sensation in the esophagus	18	8.9
	Odynophagia	3	1.5
	Cough	40	19.7
	Difficulty breathing	21	10.3
	Sialorrhea	1	0.5
	Stomach ache	19	9.4
	Hematemesis	13	6.4
	None	64	31.5
Position of the foreign body	Esophagus	124	61.1
	Intestine	22	10.
	Stomach	36	17.7
	Unidentified	21	10.3

According to Table 3, X-ray was the predominant diagnostic method used in 197 (97.0%) patients, with very few cases requiring CT (5, 2.5%) or MRI (1, 0.5%). Most cases (164, 80.8%) had no complications during the diagnostic process, though foreign body migration was observed in 14 cases (6.9%), and GI bleeding occurred in four cases (2.0%).

**Table 3:** Diagnostic techniques N=203. Examples of other complications: Temporary respiratory distress and mild allergic reaction.

Variable		N	%
Diagnostic techniques used	CT	5	2.5
	MRI	1	0.5
	X-ray	197	97.0
Complications during the diagnostic process	Perforation of the gastrointestinal tract	1	0.5
	Peritonitis	0	0.0
	Aspiration pneumonia	0	0.0
	Airway obstruction	0	0.0
	Gastrointestinal bleeding	4	2.0
	Infection	1	0.5
	Stricture formation	0	0.0
	Foreign body migration	14	6.9
	Other	2	1.0
	None	164	80.8

Table 4 shows that endoscopic removal was the most common intervention (144, 70.9%), primarily using upper GI endoscopy (139, 68.5%). Spontaneous passage occurred in 40 cases (19.7%), while surgical intervention was necessary in eight cases (3.9%). The foreign body was successfully removed in 166 cases (81.8%). Most patients (144, 70.9%) experienced no complications during management, though some cases involved foreign body migration (9, 4.4%) and various other complications such as GI tract perforation, bleeding, and infection (five cases each, 2.5%).

**Table 4:** Management and intervention N=203

Variable		N	%
Management strategy	Spontaneous passage	40	19.7
	Endoscopic removal	144	70.9
	Surgical intervention	8	3.9
	Other	11	5.4
Type of endoscope used (if appicable)	Laryngoscope	2	1.0
	Esophagoscope	6	3.0
	Upper gastrointestinal endoscope	139	68.5
	Lower gastrointestinal endoscope	2	1.0
	Not used	54	26.6
Successful removal	Yes	166	81.8
	No	37	18.2
Complications or adverse events during the management procedure	Perforation of the gastrointestinal tract	5	2.5
	Peritonitis	0	0.0
	Aspiration pneumonia	0	0.0
	Airway obstruction	1	0.5
	Gastrointestinal bleeding	5	2.5
	Infection	5	2.5
	Stricture formation	2	1.0
	Foreign body migration	9	4.4
	Surgical complications (in cases requiring surgical intervention)	1	0.5
	None	144	70.9

The outcome analysis in Table 5 showed complete resolution with medical intervention in 143 cases (70.4%) and without medical intervention in 50 cases (24.6%). Only five cases each (2.5%) had partial resolution or persistence of symptoms.

**Table 5:** Outcomes N=203

Variable		N	%
Clinical outcomes	Complete resolution with medical intervention	143	70.4
	Complete resolution without medical intervention	50	24.6
	Partial resolution	5	2.5
	Persistence of symptoms	5	2.5

Analysis of potential risk factors revealed that playing with small objects was the most common risk factor, reported in 172 cases (84.7%). This was followed by a lack of supervision, which was identified in 153 cases (75.4%). Oral exploration habits were present in 35 cases (17.2%), while developmental or behavioral disorders were noted in only 10 cases (4.9%). Other miscellaneous risk factors were reported in 16 cases (7.9%), as shown in Table 6.

**Table 6:** Potential risk factors N=203. Examples of other risk factors: Improper storage and home environment.

Variable	N	%
Playing with small objects	172	84.7
Lack of supervision	153	75.4
Oral exploration habits	35	17.2
Developmental or behavioral disorders	10	4.9
Other	16	7.9

According to Table 7, statistical analysis revealed a significant association between playing with small objects and emergency presentation (p=0.003). Among children who played with small objects, 101 (58.7%) presented as emergency cases, compared to only nine (29.0%) of those who did not. Children aged 3-5 years showed the highest rate of emergency presentations (58.9%), followed by those ≤2 years (52.3%) and children >5 years (47.9%). However, these age-related differences were not statistically significant (p=0.463). Similarly, while male children had a slightly higher rate of emergency presentations (57.5%) compared to females (42.5%), this gender difference was also not statistically significant (p=0.316).

**Table 7:** Association between demographic characteristics and risk factors N=203. Chi-square and Fisher-Freeman-Halton exact tests were used. *Association is significant at p-value <0.05.

Variable		Emergency case					
		Yes		No			
		N	%	N	%	p-value	^x2^
Age (year)	≤2	34	52.3	31	47.7	0.463	1.654
	3-5	53	58.9	37	41.1%		
	>5	23	47.9	25	52.1		
Gender	Male	69	57.5	51	42.5	0.316	1.298
	Female	41	49.4	42	50.6		
Guardian relationship to the patient	Parent	109	54.5	91	45.5	0.209	2.772
	Grandparent	1	100.0	0	0.0		
	Nurse	0	0.0	2	100.0		
Playing with small objects	Yes	101	58.7	71	41.3	0.003*	9.326
	No	9	29.0	22	71.0		
Lack of supervision	Yes	83	54.2	70	45.8	1.00	0.001
	No	27	54.0	23	46.0		
Developmental or behavioral disorders	Yes	3	30.0	7	70.0	0.191	2.479
	No	107	55.4	86	44.6		
Oral exploration habits	Yes	18	51.4	17	48.6	0.852	0.130
	No	92	54.8	76	45.2		
Other risk factors	Yes	4	25.0	12	75.0	0.018*	5.960
	No	106	56.7	81	43.3		

## Discussion

Foreign body ingestion remains a prevalent pediatric issue, presenting a significant health risk and considerably burdening the healthcare system. It has been found that 75% of cases are under four years of age, comparable to our study, where about three-fourths of the children belong to the under-five age groups [16]. A study published in 2023 by V N et al. indicates that stapler pins were the most frequently ingested foreign objects by children, accounting for 20% of cases [17]. Al Ghadeer et al. revealed that half of the patients were under the age of three years, and 62.2% were males [3]. This age and gender distribution over international and regional studies highlights the universal nature of the risk among young children. In our study, most children were brought by their parents, a finding consistent with Mazumder et al. [18].

Coins were identified as the most commonly ingested objects in our study (50.2%), a finding that mirrors those in numerous studies. Jayamaha et al. documented that coins are the most frequently (80%) swallowed foreign bodies in children [19]. Another study by Lee et al. also mentioned that coins are the most commonly ingested foreign bodies [5]. A Saudi study revealed similar findings, with coins being the most ingested foreign body (22.9%) by Saudi children [20]. The high incidence of coin ingestion can be due to frequent presence in households and their size, which makes them easily accessible and swallowable by young children. The ingestion of batteries constituted 18.2% of cases in our study, the second-most common object identified, consistent with a regional study by Ibrahim et al., in which button batteries are the second most (19.5%) ingested foreign bodies [20]. Similarly, the incidence of battery ingestion in other countries also increases over time, with a risk of being misdiagnosed as a coin, leading to complications [5,21]. The higher prevalence could be due to the increasing trend of electronic devices and the lack of safety regulations regarding battery compartments. Batteries carry a significant risk of causing severe esophageal injuries, including burns, ulcers, and corrosions in the esophageal mucosa [5]. This highlights the need for public awareness and preventive measures.

Foreign body aspiration and ingestion typically progress in three phases: the initial phase with choking and coughing, followed by an asymptomatic phase when the foreign body lodges [22]. Complications such as pneumonia, abscesses, or perforation occur in the final stage, depending upon the object's location [22]. The presentation can be divided into asymptomatic (31.5%) and symptomatic (68.5%) cases, with vomiting (48.8%) being the most common symptom. These findings are consistent with a study by Khorana et al., in which 44.3% of patients were asymptomatic and 55.7% symptomatic, with vomiting (23.2%) being the most frequently reported symptom [23]. Other common symptoms, including dysphagia and sensation of something, are also consistent with the study mentioned above. However, the significant number of asymptomatic cases highlights the need for a high level of suspicion, particularly when ingestion is witnessed or strongly suspected, even in the absence of clinical symptoms, as documented in a study at Makkah where 98.4% of patients were asymptomatic [24]. Most foreign bodies pass through the GI tract spontaneously, but those that become impacted are primarily located in the esophagus, with 75% in the upper esophagus, which aligns with our study, where 61.1% of objects were identified in the esophagus [16]. Factors affecting the spontaneous passage of the coin depend upon the location in the esophagus, age of the child, and size of the coin [5]. Radiography has a key role in diagnosing ingested foreign bodies, specifically radiopaque objects. We used radiographs to detect foreign bodies in 97% of cases, which is consistent with international guidelines [25]. CT is used to detect foreign bodies within tissues and is merely required, as in our study, it was used only in 2.5% of cases [25]. CT and MRI are used when inconclusive radiographs or complications are suspected.

Flexible endoscopy is the treatment of choice, with a success rate of 95% [26]. Our management plan includes endoscopy (70.9%), particularly upper GI endoscopy (68.5%), with an 81% success rate in esophageal foreign bodies following the international guidelines from the North American Society for Pediatric Gastroenterology, Hepatology and Nutrition (NASPGHAN), which recommends endoscopy as the first-line intervention for esophageal foreign bodies [9]. A Korean study also revealed a 98.9% success rate of endoscopic removal of the foreign body [27]. Garrido et al. recommended scoping rather than waiting [28]. In a Saudi study by Ibrahim et al., the success rate of endoscopic treatment was 98.3% [20]. Surgical removal may be required if the foreign body passes beyond the stomach and remains impacted for one week [29]. The necessity for surgical intervention was also low (3.9%) in our study. The low rate of surgical intervention highlights the effectiveness of endoscopic techniques and the importance of early intervention.

The ingestion of small objects is the leading cause of foreign body ingestion in children [30]. Our study also identified playing with small objects (84.7%) and lack of supervision (75.4%) as significant risk factors for foreign body ingestion. These results emphasize the critical role of environmental safety and parental supervision in prevention.

This study provides critical insights into patterns of foreign body ingestion in children and presents practical prevention strategies for healthcare providers and caregivers. The findings prove especially valuable for improving patient care and developing effective preventive measures.

Based on our study findings, we suggests the following evidence-based preventive measures: Healthcare providers should incorporate foreign body ingestion awareness into standard pediatric wellness visits, delivering targeted guidance to caregivers. We propose implementing comprehensive public health initiatives focused on the secure storage of high-risk items, particularly coins and batteries. Additionally, we recommend mandating enhanced safety features for battery compartments in consumer electronics. Furthermore, we advise healthcare facilities to develop and maintain robust, evidence-based protocols for managing foreign body ingestion cases, with specific emphasis on high-risk objects such as button batteries.

This study has several limitations. First, being a single-center study, the results may not be fully generalizable to other populations or healthcare settings. Second, the retrospective nature of the study may have led to incomplete documentation of some cases. Third, the study did not assess the socioeconomic factors that might influence foreign body ingestion incidents. Finally, the study period might not have captured seasonal variations in foreign body ingestion patterns.

## Conclusions

This study highlights the significant prevalence of foreign body ingestion among pediatric patients, particularly in children under five years of age, with a slight male predominance, though statistically not significant. Coins emerged as the most commonly ingested objects (50.2%), followed by batteries (18.2%). The study identified playing with small objects and lack of supervision as key risk factors. While many cases were managed successfully through endoscopic intervention (70.9%), the findings emphasize the critical importance of prevention through parental education and enhanced safety measures. The high success rate of endoscopic management (81%) demonstrates the effectiveness of current treatment protocols. However, the need for continued vigilance and prompt intervention remains paramount, especially in cases involving high-risk objects such as button batteries.
